# Supplementary material for: Modelling the impact of climate change on the distribution and abundance of tsetse in Northern Zimbabwe
Source: Parasit Vectors. 2020 Oct 19;13:526. doi: 10.1186/s13071-020-04398-3 (PMC7574501; doi:10.1186/s13071-020-04398-3)
Supplement: Supplementary file 1 — Additional file 1: Figure S1. Data for individual weather stations used to assess conversion from land-surface temperature to air-temperature. [file 13071_2020_4398_MOESM1_ESM.pdf]

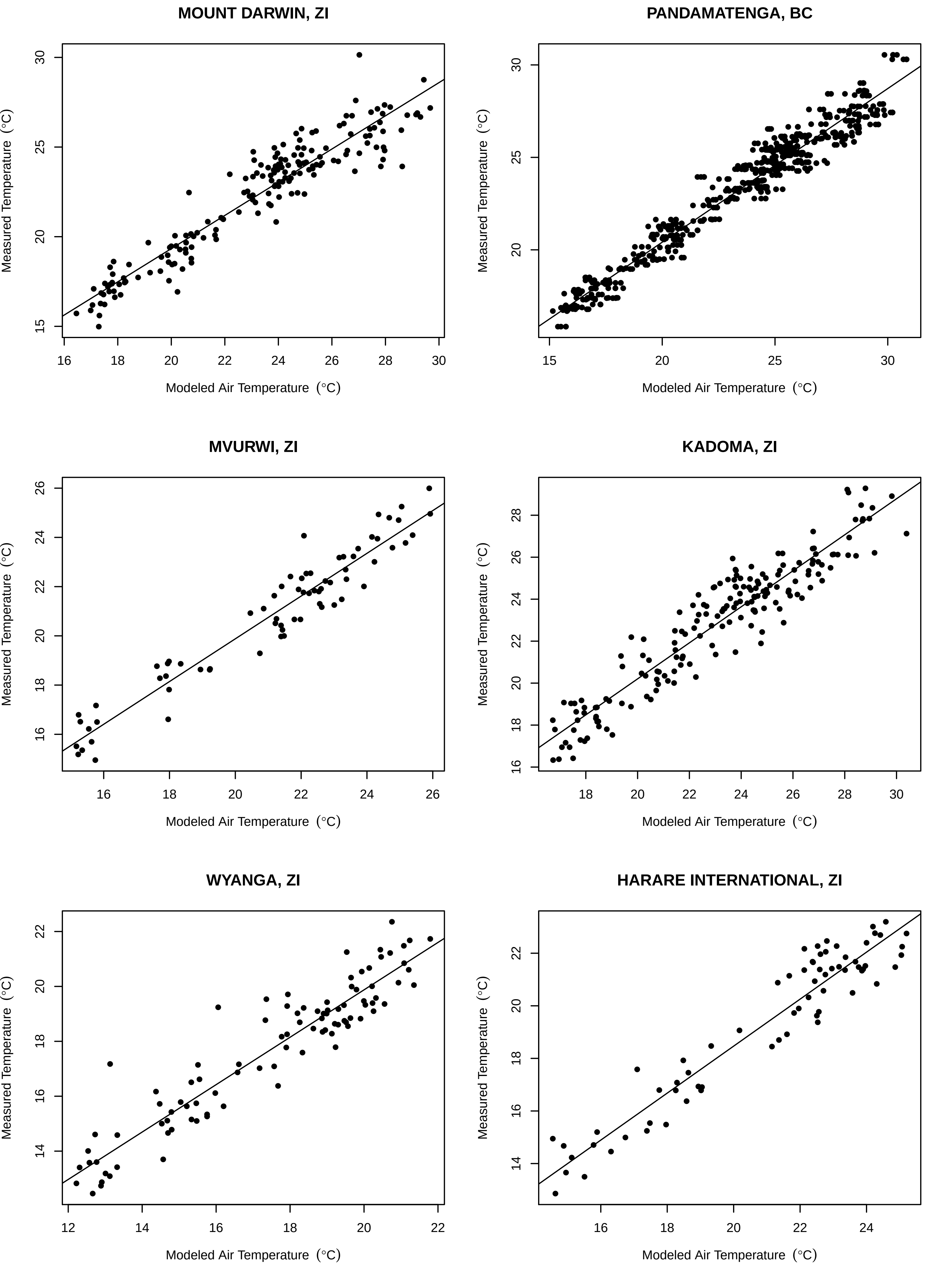
MOUNT DARWIN, ZI
PANDAMATENGA, BC
MVURWI, ZI
KADOMA, ZI
WYANGA, ZI
HARARE INTERNATIONAL, ZI
Measured Temperature (°C)
Modeled Air Temperature (°C)
15 20 25 30
16 18 20 22 24 26 28 30
15 20 25 30
16 18 20 22 24 26
16 18 20 22 24 26 28
14 16 18 20 22
12 14 16 18 20 22
14 16 18 20 22
16 18 20 22 24

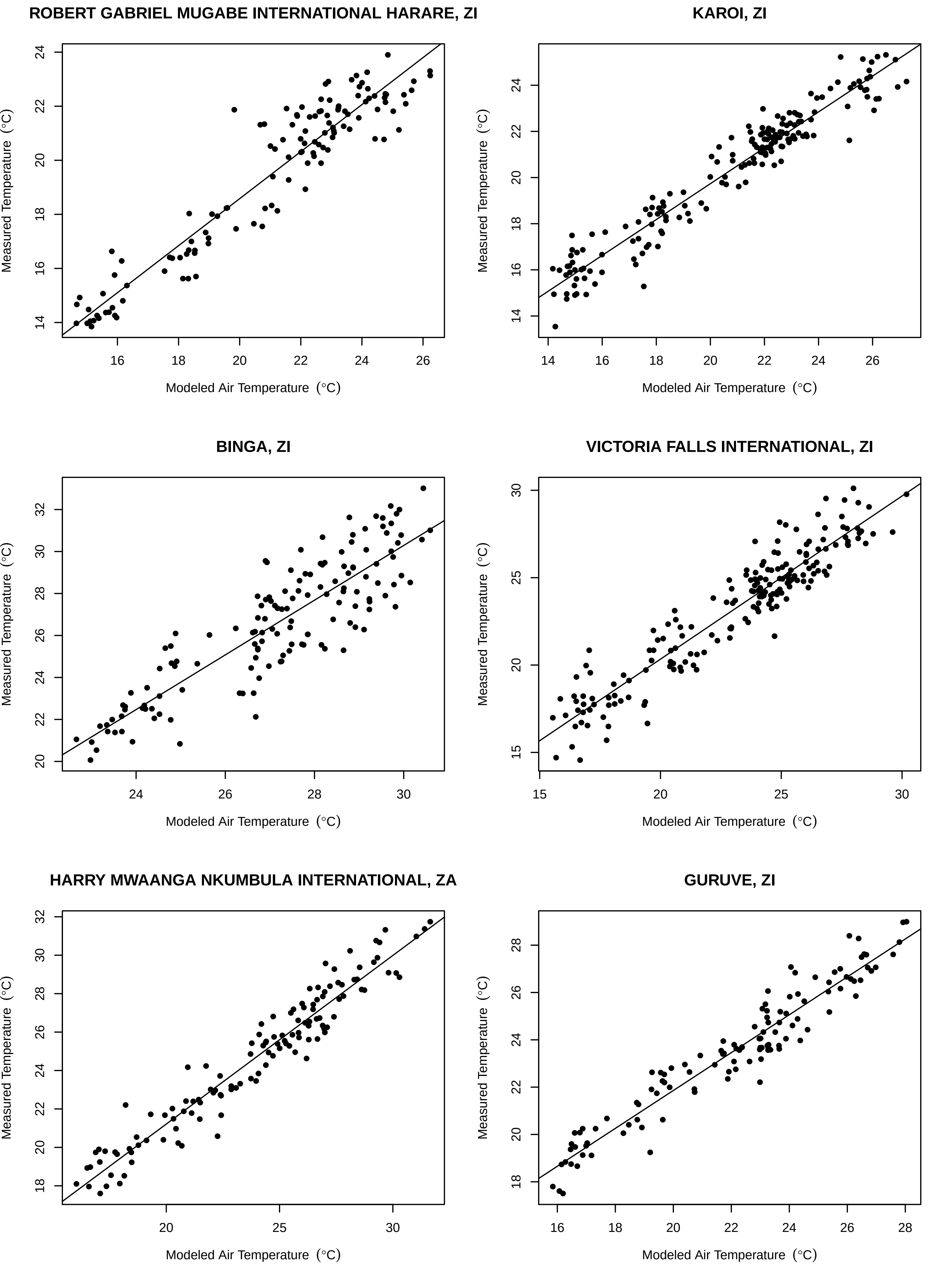
ROBERT GABRIEL MUGABE INTERNATIONAL HARARE, ZI
KAROI, ZI
BINGA, ZI
VICTORIA FALLS INTERNATIONAL, ZI
HARRY MWAANGA NKUMBULA INTERNATIONAL, ZA
GURUVE, ZI
Measured Temperature (°C)
Modeled Air Temperature (°C)

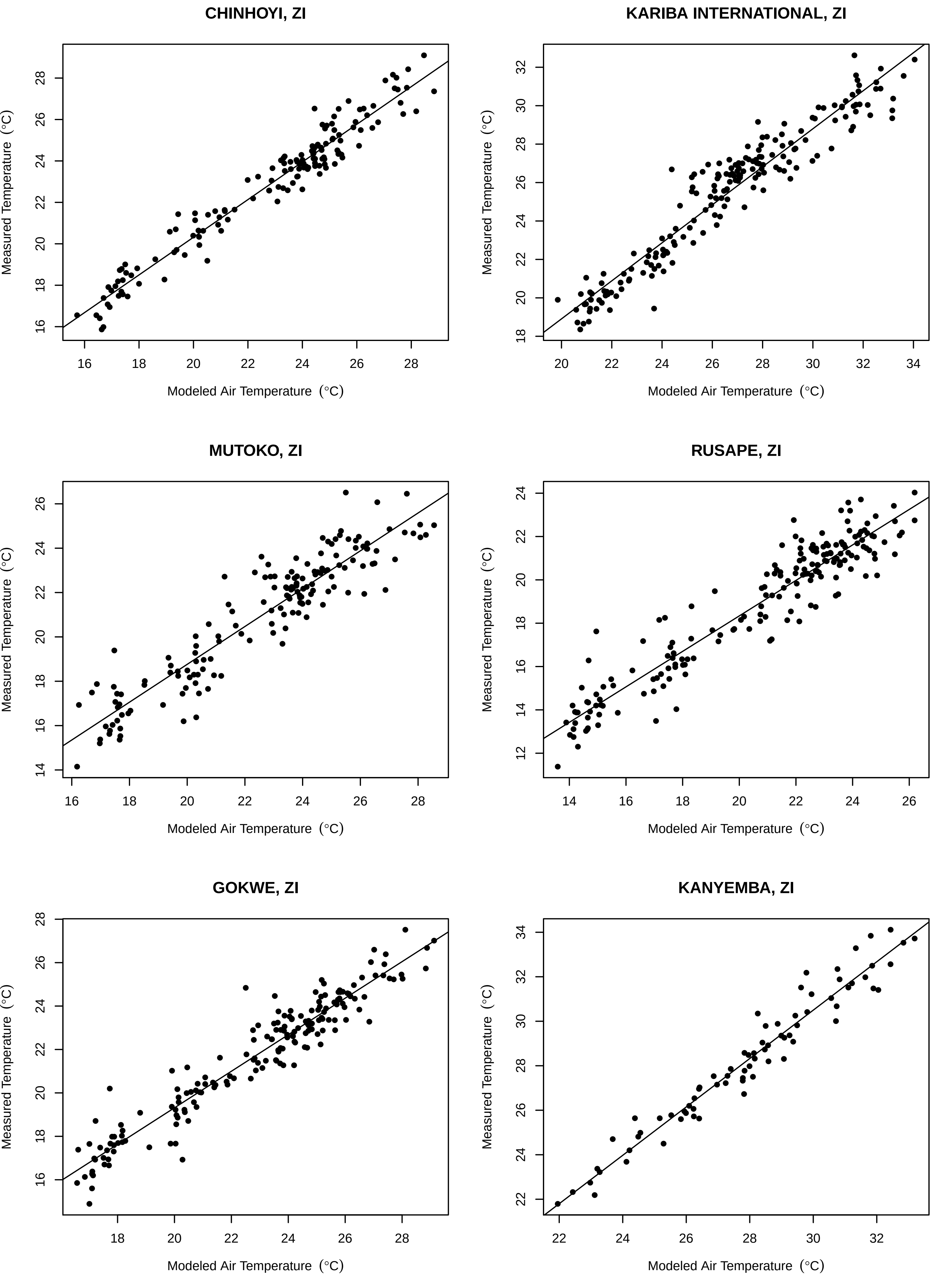
CHINHOYI, ZI
KARIBA INTERNATIONAL, ZI
MUTOKO, ZI
RUSAPE, ZI
GOKWE, ZI
KANYEMBA, ZI
Measured Temperature (°C)
Modeled Air Temperature (°C)

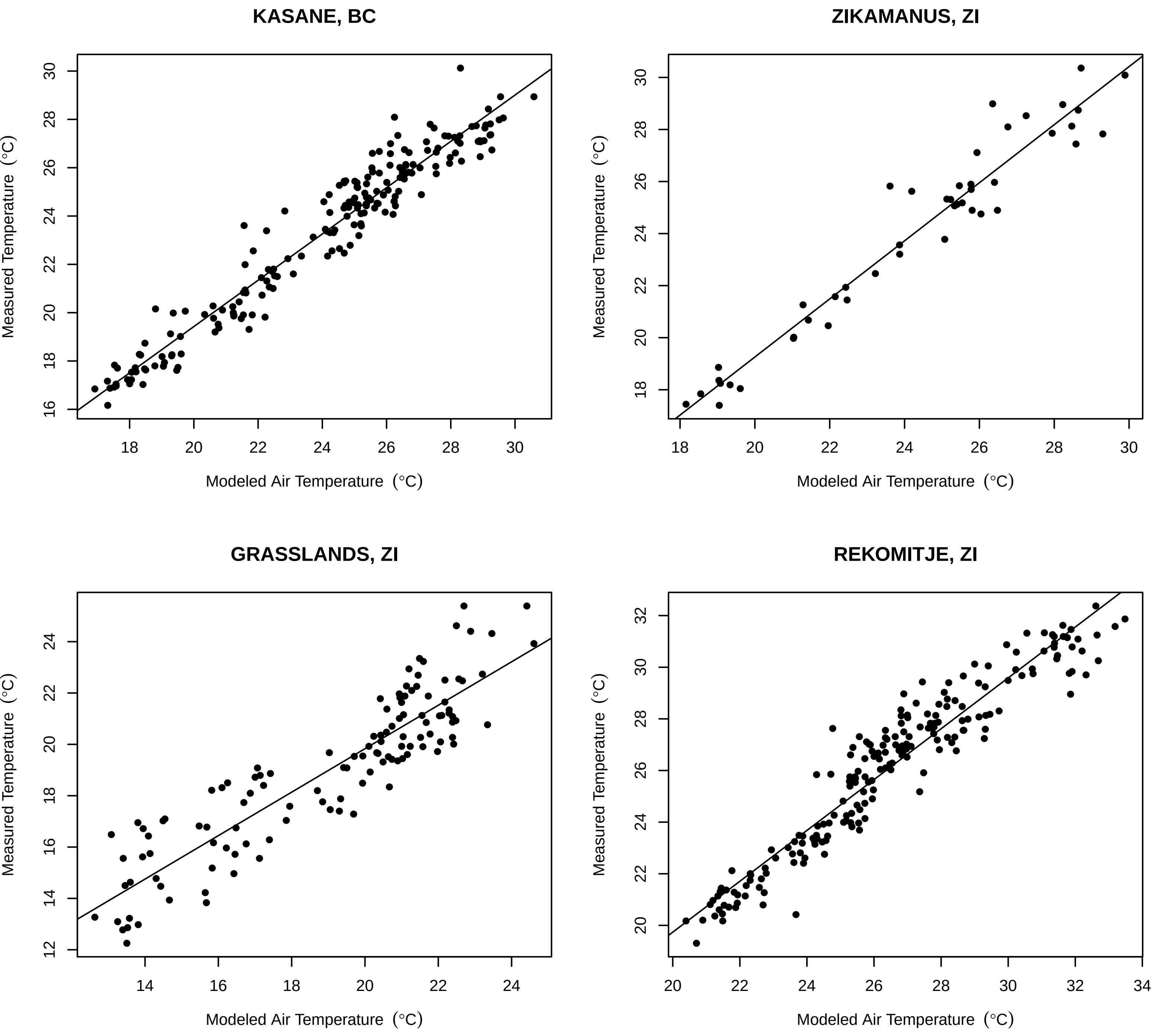
KASANE, BC
ZIKAMANUS, ZI
GRASSLANDS, ZI
REKOMITJE, ZI
Measured Temperature (°C)
Modeled Air Temperature (°C)
16
18
20
22
24
26
28
30
12
14
32
34
