# Supplementary material for: Modelling the impact of climate change on the distribution and abundance of tsetse in Northern Zimbabwe
Source: Parasit Vectors. 2020 Oct 19;13:526. doi: 10.1186/s13071-020-04398-3 (PMC7574501; doi:10.1186/s13071-020-04398-3)
Supplement: Supplementary file 4 — Additional file 4: Figure S3. Relative difference between estimated abundance (2001–2005 vs 2012–2016). Dark blue indicates areas of decrease in predicted abundance, whereas red indicates areas of a predicted increase in abundance. Areas of no change (either stable populations, or non-suitable environments) are shown in pink. Top) Simulations ran using MODIS air-temperature with an offset of -1.094 °C; Middle) Simulations ran using mean MODIS air-temperature; Bottom) Simulations ran using MODIS air-temperature with an offset of +1.094 °C. [file 13071_2020_4398_MOESM4_ESM.pdf]

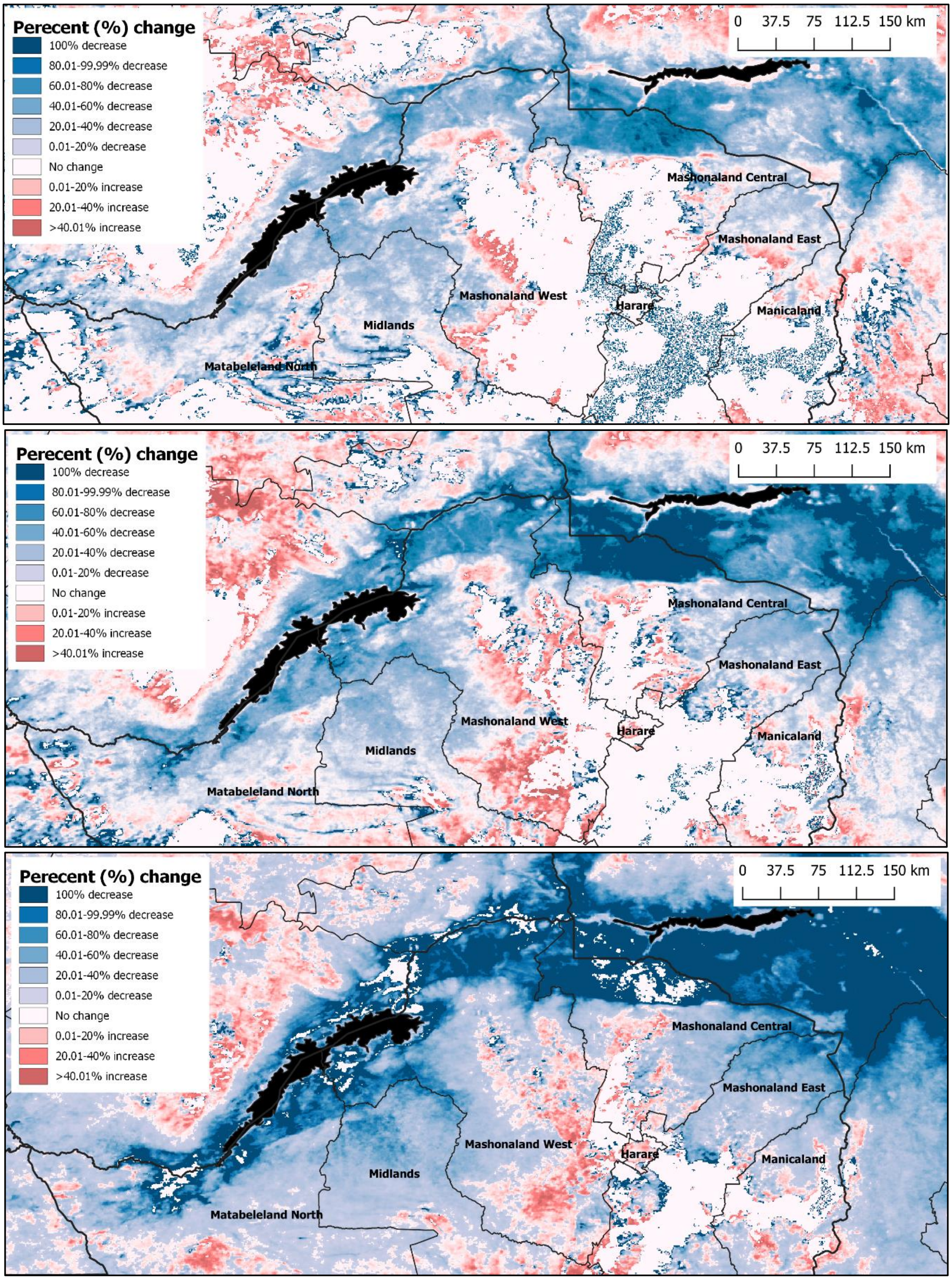

Additional file 4: Figure S3. Relative difference between estimated abundance (2001-2005 vs 2012-2016). Dark blue indicates areas of decrease in predicted abundance, whereas red indicates areas of a predicted increase in abundance. Areas of no change (either stable populations, or non-suitable environments) are shown in pink. Top) Simulations ran using MODIS air-temperature with an offset of -1.094°C; Middle) Simulations ran using mean MODIS air-temperature; Bottom) Simulations ran using MODIS air-temperature with an offset of +1.094°C.
